# Autonomous actions of the human growth hormone long-range enhancer

**DOI:** 10.1093/nar/gkv093

**Published:** 2015-02-06

**Authors:** Eung Jae Yoo, Christopher D. Brown, Yu-Cheng Tsai, Nancy E. Cooke, Stephen A. Liebhaber

**Affiliations:** 1 Department of Genetics, Perelman School of Medicine University of Pennsylvania, Philadelphia, PA 19104, USA; 2 Department of Medicine, Perelman School of Medicine University of Pennsylvania, Philadelphia, PA 19104, USA

## Abstract

The *human growth hormone* (*hGH*) gene is controlled by a long-range enhancer, HSI, located 14.5 kb 5′ to the *hGH* promoter. HSI establishes a domain of noncoding transcription that is ‘looped’ to the *hGH* promoter as an essential step in initiating *hGH* gene expression. Thus, defining how HSI generates its domain of noncoding transcription is central to understanding its long-range function. Here, we demonstrate that activation of noncoding transcription reflects an HSI-autonomous activity fully independent of interactions with linked gene promoters and occurring in spatial and temporal synchrony with initiation of *GH* expression in the embryonic pituitary. HSI establishes its noncoding transcription start sites (TSS) over a defined distance from its core determinants and in a manner independent of local primary sequences. The interval between HSI and it TSS co-maps with a domain of disordered and/or highly mobile nucleosomes specific to the pituitary locus. Thus, a localized chromatin reconfiguration by HSI and consequent establishment of an adjacent domain of noncoding transcription constitute initiating events in long-range enhancer function within the *hGH* locus.

## INTRODUCTION

The fidelity of mammalian development is critically dependent on levels, timing, and spatial positioning of gene transcription. To a great extent these transcriptional controls are mediated by a broad array of enhancer elements. The spacing between enhancers and their cognate promoters varies greatly in mammalian species, can be substantial when measured in the linear genome, and can bridge multiple unrelated loci (1). Recent studies reveal that the extensive linear separation between an enhancer and its target promoter can be overcome *via* 3D reconfigurations (‘looping’) within a chromatin locus (2–4). How such chromatin reconfiguration is initiated and stabilized and how it factors temporally and functionally into the overall pathway of enhancer actions remain to be more fully defined.

Genome-wide analyses reveal that enhancers often co-map with sites of RNA polymerase III (PolII) recruitment and noncoding transcription (5,6). The extent to which this enhancer-linked noncoding transcription contributes to target gene activation remains unclear. Functions that have been proposed for this noncoding transcription include PolII-induced nucleosome repositioning (7), delivery of PolII to a promoter *via* ‘tracking’ or ‘looping’ (8,9), and direct actions of the noncoding RNA on target gene expression (10). In some cases, enhancer-linked transcription generates long intergenic noncoding RNAs (lincRNAs) that may contribute to enhancer function(s) by acting in *cis* and/or in *trans* (10–12). Importantly, temporal and functional linkages among enhancer-dependent noncoding transcription, chromatin reconfiguration and target gene activation remain unclear in most settings.

**Figure 1.**
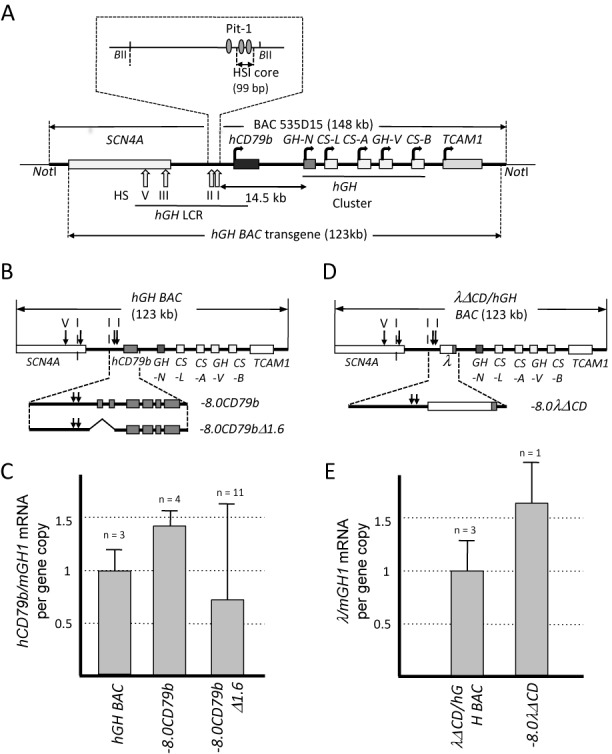
HSI activates noncoding transcription independent of promoter interactions. (**A**) Map of the *hGH* locus and the *hGH BAC* transgene. Each structural gene in the *hGH* locus is indicated along with its transcriptional orientation. The 123 kb *hGH BAC* transgene released from the originating BAC clone (BAC535D15) by NotI digestion was used to generate the *hGH BAC* transgenic mouse lines (26). The vertical arrows labeled with roman numerals indicate the positions of DNase I hypersensitive sites (HS) that form in pituitary chromatin and constitute the *hGH* LCR. The distance (14.5 kb) between the 3′ end of the HSI core and the *hGH-N* gene promoter is indicated with double-headed arrow. Note that this region encompasses the *hCD79b* gene that encodes the B-cell specific Ig receptor component, Igβ. Pit-1 binding sites that constitute core determinants of HSI are indicated by the shaded ovals (expanded inset). The 99 bp deletion that inactivates HSI functions (18) is also shown in expanded view (inset above). (**B**) Map of the wild-type *hGH BAC* and two derivative transgenes. The −*8.0CD79b* transgene (12.5 kb) encompasses HSI and the contiguous *hCD79b* (expanded view below the *hGH* locus map) and isolates this region from the *hGH* gene cluster. The *−8.0CD79bΔ1.6* transgene was derived from the *−8.0CD79b* by deletion of a 1.6-kb internal fragment extending from −0.5 kb of the promoter through intron 2, removing all defined *hCD79b* promoter elements. (**C**) Noncoding transcription across *hCD79b* in the transgenic mouse pituitary is preserved in the absence of defined promoter elements. Transcription across the *hCD79b* region generated by the promoterless −*8.0CD79bΔ1.6* was compared to that of the −*8.0CD79b* transgene (containing the intact *hCD79b* promoter but not the *hGH-N* promoter) and the *hGH BAC* transgene (containing both the *hCD79b* and the *hGH-N* promoters). Pituitary RNA from mice carrying each of these indicated transgenes was assayed for transcription across *hCD79b* by RT-qPCR. The data shown in the histogram (Y-axis) have been normalized to the corresponding transgene copy numbers and to somatotrope-specific levels of the endogenous mouse growth hormone (*mGH1*) mRNA. The number of genetically distinct lines assessed for each transgene is noted above the corresponding bars in the histogram. Each line was tested in triplicate using three independent mice. Values represent the average ± SD. The level of *hGH BAC* was defined as 1.0. (**D**) Map of the 123 kb *λΔCD/hGH* transgene and the derivative the *−8.0λΔCD* transgene. This transgene was generated by replacing the *hCD79b* gene and promoter (to −500 bp) with a size-matched fragment of bacteriophage λ DNA within the context of an otherwise intact *hGH BAC* (see ‘Materials and Methods' section). The *−8.0λΔCD* transgene was released from the *λΔCD/hGH* transgene with boundaries as indicated. (**E**) Noncoding transcription is activated 3′ of HSI within an inserted (λ) DNA segment. Transcription across the λ-DNA region was compared between the *λΔCD/hGH* and *−8.0λΔCD* transgenes. λ noncoding transcription was measured by RT-qPCR, normalized to transgene copy-number and to *mGH1* mRNA levels. λ noncoding transcription was actively transcribed without *hGH-N* promoter. The number of genetically distinct lines assessed for each transgene is noted above the corresponding bars in the histogram. Each experiment was carried out in triplicate. The study of the single *−8.0λΔCD* line was evaluated in each of three independent mice, each studied in triplicate. Values represent the average ± SD.

The *human growth hormone* (*hGH*) gene is under the control of a long-range enhancer. This enhancer, HSI, constitutes the major pituitary-specific transcriptional determinant within the multicomponent *hGH* locus control region (LCR) (Figure 1A) (13). HSI is located at the 3′ boundary of the *hGH* LCR, 14.5 kb 5′ to the *hGH-N* promoter (Figure 1A). It is flanked 5′ by a second pituitary specific HS, HSII, that lacks intrinsic enhancer activity (14). HSI was initially identified by DNaseI mapping of somatotrope-enriched human pituitary tumors and was subsequently demonstrated to be faithfully modeled in mice carrying extensive *hGH* transgene loci (14–16). *In vivo* analyses in transgenic mouse models subsequently revealed that the core component of HSI comprises an array of binding sites for the pituitary-specific master-regulatory transcription factor Pit-1 (POU1-F1) (Figure 1A) (17). Pit-1 binding at HSI triggers extensive histone modification throughout the *hGH* locus (18) and is essential for bringing HSI into close proximity (‘looping’) with the *hGH-N* promoter (19). Deletion of HSI Pit-1 binding sites results in loss of HSI formation and a 20-fold decrease in *hGH* expression (18). Thus, HSI activation of *hGH-N* expression represents a well-defined and experimentally tractable model for the *in vivo* analysis of long-range enhancer functions.

The initial mapping of the *hGH* locus revealed that a B-cell specific gene, *hCD79b*, is situated between HSI and the *hGH-N* promoter (Figure 1A) (20). *hCD79b* encodes the Igβ protein, an essential subunit of the B-cell receptor (21). Igβ is specific to B cells and is not detected in pituitary (22). It was therefore surprising to observe robust expression of *hCD79b* mRNA in primary human somatotropes and in mouse pituitaries carrying an *hGH* transgene (22). While initial studies suggested that this *hCD79b* mRNA might reflect non-functional ‘bystander’ transcription (22), subsequent analyses demonstrated that this noncoding transcriptional activity plays an essential role in *hGH* activation in the pituitary somatotrope (19,23). Thus, understanding how HSI activates this noncoding domain is central to delineating mechanisms underlying its long-range enhancer activity and *hGH-N* transcriptional control(s).

*hCD79b* transcription in B cells and in the pituitary are under distinct regulatory controls. In B cells, *hCD79b* transcription is dependent on the interaction of B-cell specific transcription factors with promoter and promoter-proximal determinants (24). Deletion of these determinants ablates *hCD79b* expression in B cells but has no impact on transcription across *hCD79b* in the pituitary (22). In a reciprocal manner, transcription of *hCD79b* in the pituitary is uniquely dependent on HSI function (22,23); targeted inactivation of HSI ablates *hCD79b* transcription in the pituitary but has no impact on *hCD79b* transcription in B cells (25). Importantly, the direct blockade of transcription across *hCD79b* in the pituitary represses *hGH-N* expression without altering HSI formation (19). These data demonstrate a direct role of the HSI-dependent noncoding transcription in HSI enhancer function. Importantly, this role reflects the noncoding transcriptional activity *per se*, rather than the structure of the locally generated noncoding RNA (23). These observations support a model in which HSI activation of noncoding transcription constitutes an initiating event in the pathway of *hGH* locus activation (23).

In the present study, we explore the mechanisms by which HSI activates its adjacent domain of noncoding transcription. Our studies reveal that this HSI activity is autonomous of interactions of HSI with linked promoters. The activation of this HSI-dependent noncoding transcriptional activity is further shown to be developmentally appropriate, tracking both spatially and temporally with the initiation of *mGH* expression in the primordial mouse pituitary. Mapping of the corresponding noncoding transcriptional start sites (TSS) reveals that they are positioned 1.6 ± 0.2 kb 3′ to the HSI core determinants. This intervening distance co-maps to a domain of disordered and/or highly mobile nucleosomes specific to the pituitary. These data support a model in which HSI reconfigures local chromatin structure to support a robust set of noncoding TSSs. These HSI activities are proposed to constitute initiating events in its long-range enhancement of *hGH-N* gene expression.

## MATERIALS AND METHODS

### Generation of transgenic mouse lines

Transgenic lines were generated by microinjecting purified transgene DNAs into fertilized mouse oocytes (C57BL/6XSJL) by Transgenic and Chimeric Mouse Facility (TCMF) of the University of Pennsylvania. Founders were identified by PCR of tail DNA and transgene copy numbers were determined by Southern blot analyses of purified genomic DNA digested with NcoI; a 3.5-kb *hCD79b* fragment and a 1.6-kb *mζ-globin* fragment were hybridized using corresponding α-dCTP-labeled probes. Each band signal was scanned and quantified using Typhoon FLA9500 Phosphorimager (GE Healthcare) and the normalized ratio was used to establish the transgene copy number. The transgenic lines *hGH BAC, λΔCD/hGH BAC, CDΔ0.7/hGH BAC, CDΔ1.6/hGH BAC* and −*8.0CD79b* have been described previously (23,25,26).

### RNA isolation and real-time RT-PCR (qPCR)

Total RNA was extracted from mouse pituitaries with RNA-Bee (Tel-Test), treated with RQ DNase (Promega) at 37°C for 1 h, and purified using an RNeasy Minikit (Qiagen). In all cases, 1 μg of purified RNA was reverse-transcribed using a High-Capacity cDNA Reverse Transcription Kit (Applied Biosystems) and RNA levels were assessed by real-time PCR using TaqMan Universal PCR Master mix (Applied Biosystems) and a 7900HT platform with corresponding probes (*hCD79b*, Hs00236881_m1; λ; *mGAPDH*, Mm99999915_g1). SDS2.4 software was used for analyses.

### *In situ* hybridization

*mPit-1, mGH1* and *hCD79b* cDNAs were individually amplified by RT-PCR from purified pituitary RNA (primers listed in Supplementary Table SI) and cloned into the pGEM-T easy vector (Promega). DIG-labeled anti-sense RNA probes were generated by SP6 or T7 *in vitro* transcription, treated with DNase I (New England Biolabs), precipitated with ethyl alcohol, dissolved in TE buffer (10 mM Tris pH8.0, 1 mM ethylenediaminetetraacetic acid (EDTA)), and stored at −80°C until use. Embryos from defined transgenic lines were collected at specified days post coitum (dpc) and fixed overnight at 4°C in 4% paraformaldehyde in phosphate buffered saline (PBS). The fixed embryos were immersed in 30% sucrose in PBS at 4°C, embedded in OCT (Sakura), and quick-frozen on dry ice. Twenty micrometers slices were generated by cryostat and *in situ* hybridizations were performed as described by Nissim et al. (27).

### 5′ Rapid amplification of cDNA ends (5′ RACE analysis)

Total RNA isolated from transgenic mouse pituitaries was reverse-transcribed by Superscript III reverse transcriptase (Invitrogen) at 55°C for 1 h using a sequence-specific antisense primer (Supplementary Table SI). Free residual dNTPs were removed by G-50 Spin column (GE Healthcare), a poly G tail was added to the 3′ end of the transcribed cDNA using terminal dinucleotidyl transferase (Promega), and the tailed cDNA was then amplified between an adaptor-polyC_17_ primer and a nested primer (Supplementary Table SI). The amplified cDNAs were cloned into the pGEM-T Easy Vector (Promega) and individually sequenced (University of Pennsylvania Genomic Analysis Core).

### Isolation of mononucleosome-protected DNA fragments

To generate somatotrope-enriched mouse pituitaries, *hGH BAC* transgenic mice (1255B, containing two transgene copies) were crossed with *GRF* transgenic mice (28) and pituitaries were extracted from 3-month-old double-positive progeny. Five pituitaries were combined and dissociated using Hanks-based Cell Dissociation Buffer (GIBCO) and filtered through 40 μm nylon-mesh strainers (BD Sciences). Mouse B lymphocytes were enriched from the spleen by the Ficoll-gradient method using Ficoll-Plaque plus (Amersham) as previously reported (22) and further purified on MACS CD43 (Lys48) microbeads (Militenyi Biotec) using the MACS cell sorting kits (Militenyi Biotec). Nuclei from both tissues were isolated and prepared using EZ Nucleosomal DNA Prep Kit (Zymo Research) and nuclei in 500 μl MN digestion buffer were treated with 2 U Micrococcal Nuclease (Zymo Research) at room temperature for 10, 20 and 30 min. Digestions were terminated by adding 20 μl MN Stop Buffer (Zymo Research) to each 100 μl aliquot. Final digested DNAs were purified using Zymo Spin IIC columns (Zymo Research) according to manufacturer's instructions. The graded partial digestions were assessed for quality by analysis of purified DNAs on 2% agarose gels. The fragments corresponding to mononucleosomes (DNA fragments in the 120–200 bp size range) were isolated by Pippin electrophoretic size selection using 2% agarose gel cassette (Sage Science).

### Next generation library preparation and sequencing

The isolated DNAs corresponding to mononucleosome core fragments were end-repaired, 3′ adenylated, ligated to adaptors, and amplified using NEBNext ChIP-Seq DNA Sample Prep Reagent for Illumina kit (New England Biolabs). The oligonucleotides used for library preparation are listed on Supplementary Table SI. The concentration and size distribution of each library was validated using an Agilent Bioanalyzer (Agilent Technologies). Single-end sequencing (50 bp) was performed on each library using a HiSeq2000 by Next Generation Sequencing Core (NGSC) at the University of Pennsylvania.

### Nucleosome mapping

A chimeric genome assembly was constructed by concatenating the standard mm9 assembly with an additional human sequence contig (hg19) spanning the transgene locus (chr17:61932183–62053367). Reads were aligned to this chimeric genome using bwa-0.7.8 (Commands bwa aln –q 15 and bwa samse) (PMID 19451168). Alignments were merged and coordinate sorted using samtools-0.1.18 (PMID 19505943). Nucleosome coverage and nucleosome positioning stringency were estimated using NuMap-0.9 (default parameters, core size = 147 bp and spacing = 193 bp) (29). Coverage, stringency and dyad plots were all generated using the R package Sushi (30).

## RESULTS

### HSI activates noncoding transcription in the absence of linked promoters

We initially asked whether the domain of noncoding transcription 3′ to HSI could be established autonomously by HSI. This would contrast with extant models of noncoding transcription based on cooperative enhancer/promoter interactions (10). Our prior studies have demonstrated that HSI-dependent noncoding transcription can be fully activated on a transgene that is limited to HSI and the contiguous *hCD79b* gene (−*8.0CD79b*; Figure 1B) (23). While this demonstrates that HSI can function independent of its cognate *hGH-N* promoter, it leaves open the possibility of interactions between HSI and the more proximal *hCD79b* promoter. Thus, to further test HSI-autonomy, we introduced a 1.6-kb interstitial deletion within the *−8.0CD79b* transgene that removed the *hCD79b* promoter along with a secondary promoter located within the first intron (−*8.0CD79bΔ1.6*; Figure 1B). The removal of these *hCD79b* promoters eliminates *hCD79b* transcription in B cells, but not in pituitary (25). Remarkably, each of 11 genetically distinct −*8.0CD79bΔ1.6* transgenic lines expressed appreciable levels of *hCD79b* mRNA in the pituitary. In 10 of these lines the levels of *hCD79b* mRNA ranged between 0.2-fold to 1.7-fold of the levels generated by the intact *hGH/BAC* transgene (defined as 1.0 in this assay). A single remaining line was expressed at substantially lower levels, but still clearly above background. An inclusive compilation of these data from all 11 lines (Figure 1C) demonstrates that transcription from the promoterless −*8.0CD79bΔ1.6* transgene in the pituitary is robust. The observed line-to line-variation for −*8.0CD79bΔ1.6* transgene transcription most likely reflects the isolation of HSI from insulators and linked promoters in the native locus that shield it from surrounding transcriptional influences.

HSI autonomy was further tested by creating a second promoterless transgene in which the *hCD79b* gene and its promoter (extending to −500 bp) was replaced by an identically sized (3.6 kb) segment of bacteriophage λ DNA (*−8.0λΔCD*, Figure 1D). A single *−8.0λΔCD* transgenic line was established for this purpose and transcription across the λ-DNA segment in the pituitary was examined. Importantly, the level of transcription of this promoterless transgene was robust and was comparable to that of the full-length *λΔCD/hGH BAC* transgene locus containing the identical substitution of λ-DNA for the *hCD79b* promoter/gene (Figure 1D and E). Thus, these two sets of transgenic studies support the conclusion that HSI can activate its functionally critical domain of noncoding transcription independent of interactions with linked gene promoters.

### The HSI enhancer accurately activates noncoding transcription in the embryonic pituitary

**Figure 2.**
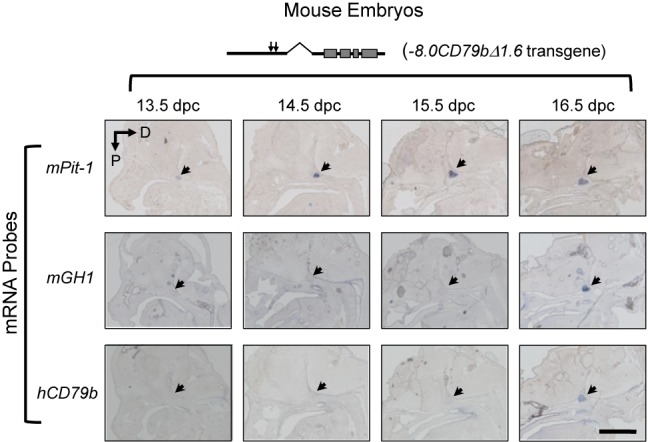
HSI-dependent noncoding transcription is appropriately activated during embryonic development. Mouse embryos carrying the promoterless *−8.0CD79bΔ1.6* transgene (Figure 1B) were assessed at 13.5 dpc through 16.5 dpc by *in situ* histohybridization. Each embryo was hybridized with antisense probes to *mPit-1, mGH1* and *hCD79b* transcripts and sections were counterstained with alkaline phosphatase-conjugated anti-DIG antibodies and with BM Purple (Roche) to reveal anatomical landmarks. The region occupied by the embryonic pituitary in each sagittal head section is indicated (arrow). The appropriate activation of m*Pit-1*, and *mGH1* at 14.5 dpc and 16 dpc, respectively, confirmed embryonic staging and pituitary positioning. Transcription across the *hCD79b* region of the promoterless *−8.0CD79bΔ1.6* transgene was observed to be in full positional and temporal synchrony with that of *mGH*. D, dorsal; P, posterior. Scale bar, 1 mm.

We next asked whether HSI can independently dictate appropriate spatial and temporal activation of noncoding transcription during embryonic development (Figure 2). Control *in situ* histohybridizations of developmentally staged −*8.0CD79bΔ1.6* embryos confirmed the expected pituitary-specific activation of the endogenous *mPit-1* and *mGH1* genes at e14.5 and e16.5, respectively. The two day lag between *Pit-1* mRNA expression and *mGH* transcriptional activation has been previously noted and ascribed to a translational delay (31). The analysis of the promoterless −*8.0CD79bΔ1.6* transgenic embryos revealed spatial and temporal specificity of transcriptional activation that was identical to that of the endogenous *mGH*. These data support the conclusion that the HSI enhancer is sufficient not only to sustain its domain of noncoding transcription in the adult pituitary, but also to activate this transcriptional domain with appropriate timing and positioning during embryonic development.

### The HSI enhancer initiates noncoding transcription at a defined distance 3′ to its core elements

**Figure 3.**
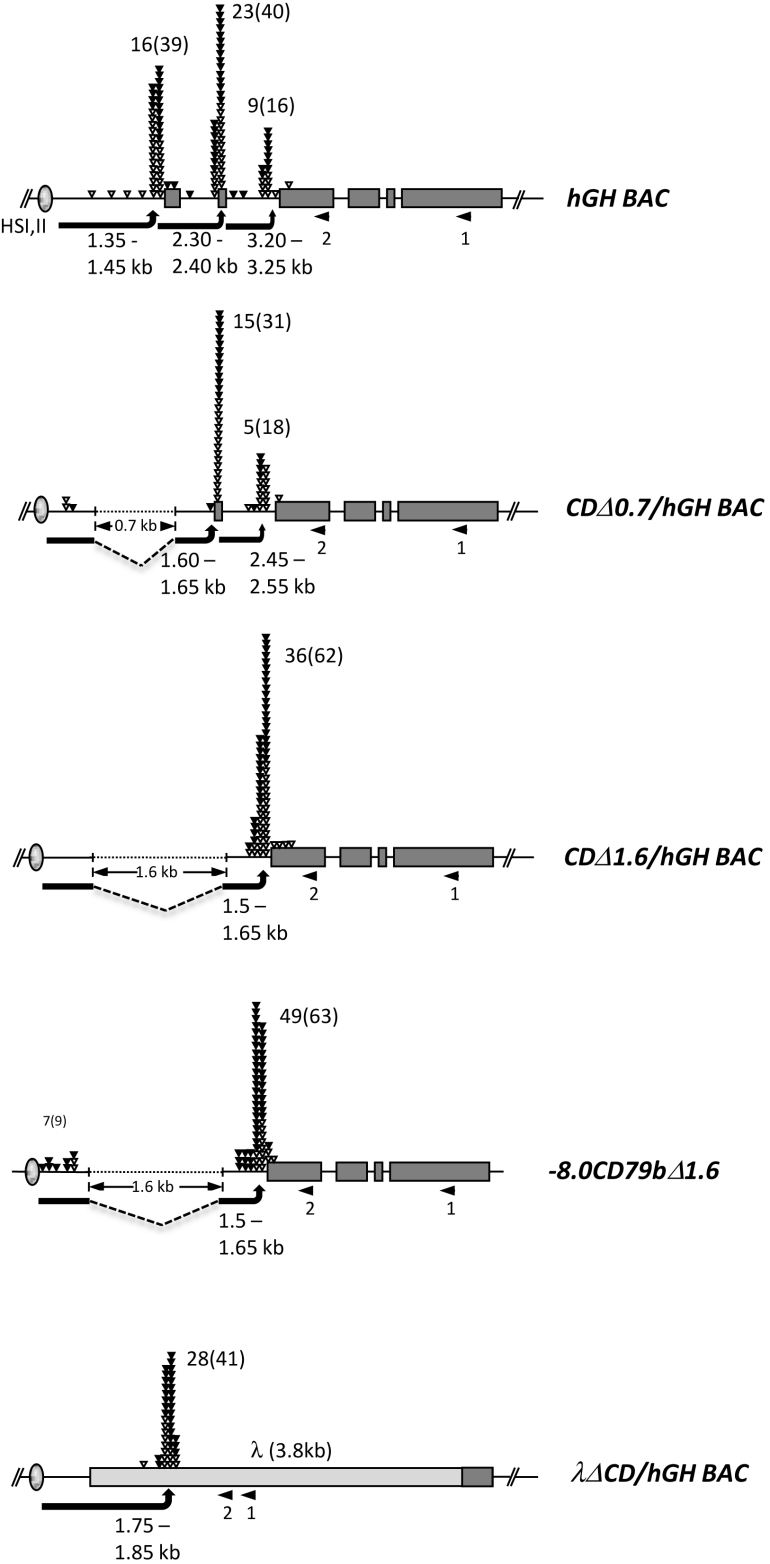
5′ RACE analyses of transcription start sites 3′ to HSI in the *hGH BAC* and derivative transgenes reveals consistent spacing between the HSI core determinants and the TSS cluster. RNA was isolated from the pituitaries of mice carrying each of the 5 indicated transgenes (Figure 1). In each case, cDNA synthesis was primed at the site labeled 1 (left facing arrow), poly G tails were added to the 3′ end of the cDNA, and the product was then amplified between an adapter-polyC_17_ primer and a nested primer (arrow 2). The amplified PCR products were cloned and individually sequenced. Each triangle indicates the 5′ terminus of an individual clone and the results were grouped within 50 bp windows. The total numbers of cDNAs containing an additional non-templated terminal G (corresponding to the 5′-capped structure; filled triangles) are shown, and the total numbers of cDNAs with and without the nontemplated G are included in parentheses. The arrows indicate the distance between the HSI core and the center of the most proximal TSS cluster. *hGH BAC*, 123 kb unmodified human transgenic mouse line (two copies) (26); *CDΔ0.7/hGH BAC*, 0.7 kb deletion of 0.5 kb promoter region and exon 1 from *hGH BAC* (14 copies) (25); *CDΔ1.6/hGH BAC*, 1.6 kb deletion of 0.5 kb promoter through exon 2 from the *hGH BAC* (three copies) (23); *−8.0CD79bΔ1.6*, 1.6 kb deletion of *hCD79b* promoter through exon 2 from the *−8.0CD79b* (6 copies); *λΔCD/hGH BAC*, 3.8 kb λ gene segment replacing *hCD79b* from *hGH BAC* (five copies) (23).

How HSI establishes adjacent noncoding transcriptional activity was next explored by mapping the 5′ boundary of the noncoding domain (see ‘Materials and Methods' section and Figures 1 and 3). cDNA segments were generated by 5′ RACE from pituitary mRNA of mice carrying a set of informative transgenes. These cDNAs were individually sequenced and their 5′ termini were grouped into 50 bp windows (Figure 3). The presence of an additional non-templated G (corresponding to the mRNA cap structure) in a substantial fraction (∼50%) of cDNAs within a window supported their generation by Pol II (filled triangles; Figure 3) (32).

TSSs located 3′ to HSI were initially mapped in the pituitaries of mice carrying the intact 123 kb *hGH BAC* transgene (Figure 1A, top). This 5′ RACE analysis revealed a major cluster at 1.35–1.45 kb and the two additional clusters located at 900 bp intervals further 3′. All three clusters contained a substantial representation of non-templated 5′ terminal C (41.0–57.5%) validating their identity as PolII initiation sites. TSS 3′ to HSI were next mapped in two *hGH BAC* transgenes containing nested deletions of the *hCD79b* promoter (Figure 3, second and third diagrams); a 700 bp deletion (*CDΔ0.7/hGH BAC*) removing the B-cell specific *hCD79b* promoter and a more extensive 1.6 kb deletion extending further 3′ through a secondary promoter located within intron 1 (*CDΔ1.6/hGH BAC*) (25). 5′ RACE of pituitary RNA from these two sets of transgenic mice revealed TSS clusters located 1.60–1.65 kb and 1.50–1.65 kb 3′ to the HSI core. 5′ RACE analysis of the promoterless (*−8.0CD79bΔ1.6*) transgene (Figure 3, fourth diagram) revealed the same distance (1.50–1.65 kb) between HSI and the TSS cluster. A final 5′ RACE analysis mapped the TSS 3′ to HSI in the *λΔCD/hGH BAC* transgene with the substitution of the *hCD79b* gene (−500 bp through the terminal exon) by an identically sized segment of λ DNA) (Figures 1D and 3, bottom diagram). The TSS cluster in this setting mapped within the λ-DNA insert at a distance of 1.75–1.85 kb 3′ to the HSI core. As in the cases of TSS within the *hCD79b* gene, approximately 50% of the λ cDNA sequences contained a 5′-terminal, non-templated G. Of note, an alignment of the primary sequences bracketing the TSS clusters within the *hCD79b* and the *λ* DNA failed to reveal elements corresponding to recognized promoter consensus sequences such as CpG rich domains or TATA boxes (Supplementary Figure S1). These data support the conclusion that HSI is able to activate noncoding TSS at a mean distance of 1.6 ± 0.2 kb 3′ from HSI and in a variety of primary sequence environments. How this particular spacing between HSI and its noncoding TSS is established was next assessed.

### The spacing between HSI and its noncoding TSS cluster co-maps with a pituitary-specific chromatin configuration

The activation of noncoding TSSs by HSI in the pituitary appears to reflect a non-canonical mechanism dependent on HSI actions. The TSS in the pituitary are solely dependent on HSI function while those in the B cells are solely dependent on *hCD79b* promoter-proximal elements (see 'Introduction' section). To explore the basis for the activation of TSS in the pituitary, we directly compared nucleosome architecture in the pituitaries and B cells of *hGH BAC* mice. Mononucleosome preparations generated from somatotrope-enriched pituitaries and splenic B cells were digested with micrococcal nuclease for 10, 20 or 30 min (Figrue 4 and ‘Materials and Methods' section). These timed digestions were designed to release mononucleosomes from ‘open’ as well as ‘compacted’ regions of the genome and to minimize any loss of DNA associated with loosely bound nucleosomes. Mononucleosome populations were gel purified, pooled and sequenced to a minimum depth of 10-fold genome coverage (Table 1). Nucleosome coverage throughout the *hGH BAC* transgene was mapped for each cell type (Figure 5A) and corresponding nucleosome positioning stringency and dyad metrics were established (Figure 5B and C).

**Figure 4.**
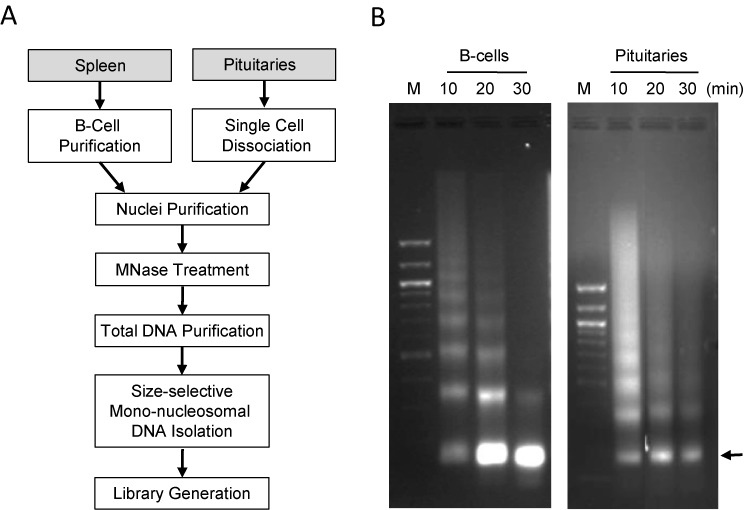
Isolation and analysis of nucleosome-protected DNA fragments from pituitaries and splenic B cells of *hGH BAC* transgenic mice. (**A**) Work flow diagram. Spleens and pituitaries from *hGH BAC* mice were isolated and B cells were enriched on antibody coated beads. Nuclei isolated from disaggregated cells were digested by MNase for 10, 20 or 30 min. DNAs corresponding to mononucleosome-protected fragments were isolated from each of these digestions and used to generate whole genome sequencing libraries (see ‘Materials and Methods' section). (**B**) Analytic gel displaying DNA isolated from MNase partial digests of the indicated chromatin preparations. The position on the 2.0% agarose gel corresponding to the mononucleosome-protected DNA fragments (∼150 bp) is indicated by the arrow.

**Figure 5.**
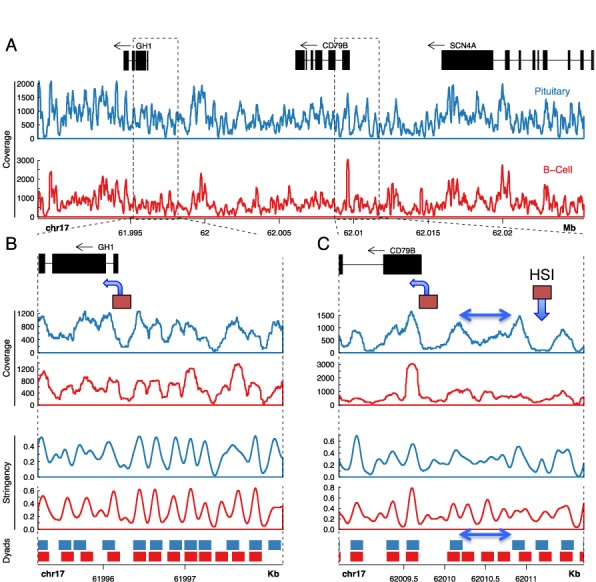
Nucleosome occupancy at the *hGH BAC* transgene locus reveals a singular chromatin configuration in the pituitary at HSI and in the region between HSI and the *hCD79b* promoter. (**A**) Sequence coverage of MNase-generated mononucleosomes across the region encompassing the *hGH-N* and *hCD79b* genes in the *hGH BAC* transgene. Mononucleosome-protected DNA generated by MNase digestions (Figure 4) from splenic and pituitary nuclei of *hGH BAC* transgenic mice were converted into NGS libraries and sequenced to a minimum 10× genome coverage (Table 1). Sequence data was mapped to the chimeric *hGH BAC* transgenic genome. Gene annotations are depicted at the top of the figure (in black). Nucleosome coverage as determined by sequencing of mononucleosomes generated by the MNase digestions (Figure 4) from pituitary and B cells are plotted below (blue and red, respectively). The genome coordinates are indicated below the coverage plots. (**B** and **C**). Expanded views of the *hGH-N* promoter region and *hCD79b* promoter-HSI region, respectively. Gene annotations are depicted in the top panel in black, and the *hGH-N* and *hCD79b* promoters are each indicated by boxes with an arrow in the direction of transcription. The HSI core region is indicated by a box and downward arrow. Pituitary and B-cell nucleosome ‘coverages’ are plotted in the first and second panels, followed by the ‘stringency’ plots in the third and fourth panels, followed by the nucleosome ‘dyad positioning’ plots in the bottom panel (as labeled). Stringency plots represent the fraction of defined nucleosome positions in a region (29). Dyad positioning plots depict regions occupied by nucleosomes at high stringency (>50%). The double-headed arrow in figure (C) represents the region of disordered nucleosomes between HSI and the *hCD79b* promoter that is specific to the pituitary locus.

**Table 1.** Depth of sequencing coverage

Libraries	10 min	20 min	30 min	Sum
Pituitary	2.966	4.512	3.111	10.589
B cells	10.644	5.464	8.220	24.328

The detailed analysis of nucleosome occupancy focused on the regions encompassing the *hGH-N* promoter and that bridging HSI and *hCD79b* (Figure 5B and C, respectively). The 2 kb region encompassing the *hGH-N* promoter (Figure 5B) contains 12 regularly spaced nucleosomes that are well aligned between the pituitary and the B-cell samples (blue and red plots, respectively). The *hGH-N* promoter itself aligns with a 200-bp nucleosome free region that is flanked by positioned nucleosomes in both pituitary and B cells although this nucleosome-free region is more pronounced in the pituitary, the site of *hGH* expression (Figure 5B). The *hCD79b* promoter co-maps in both cell types with a nucleosome-free region flanked by strongly positioned nucleosomes (Figure 5C).

In contrast to these conserved nucleosomal organizations surrounding the *hGH-N* and *hCD79b* promoters in pituitary and B cells, the chromatin structures at HSI and in the region between HSI and the *hCD79b* promoter in these two tissues are clearly divergent (Figure 5C). The HSI core aligns with a 250-bp nucleosome-free region bound by two prominent peaks in the pituitary (Figure 5C, blue). This configuration is absent in the B cells (Figure 5C, red), consistent with the pituitary-specificity of HSI formation (14). The 600 bp regions separating *hCD79b* promoter and HSI in the two tissues were next compared (Figure 5C). This region in the B cells (Figure 5C, double headed arrows) contains three well-positioned nucleosomes with canonical 200 bp spacing (Figure 5C, red; best visualized in the stringency and dyad plots). The corresponding region in the pituitary (Figure 5C, blue), demonstrates substantial levels of nucleosome-protected DNA, but lacks any discernable pattern of nucleosomal positioning. This configuration between HSI and its most proximal cluster of TSSs in the pituitary is most consistent with a region of disorganized and/or highly mobile nucleosome architecture (33).

## DISCUSSION

Recent advances in genome-wide technologies have generated significant insights into linkages between chromatin structures and gene regulation. A finding of particular interest is the association of enhancer elements with localized regions of noncoding transcription (11). While widespread, the mechanisms by which enhancers generate noncoding transcription and the ultimate biologic impact of this transcriptional activity on gene regulation remain unclear. In certain cases, it has been demonstrated that the activation of enhancer-linked noncoding transcription requires synergistic interactions of the enhancer with a cognate promoter (10). The generality of such findings, their mechanistic bases, and the anatomies of the enhancer linked noncoding transcriptional domains all remain incompletely defined.

The *hGH* gene is under the control of a defined enhancer determinant, HSI, located 14.5 kb 5′ to the target *hGH-N* promoter (13,18,34). The formation and function of HSI is strictly limited to the somatotrope lineage in the anterior pituitary (13,16) and depends on an array of binding sites for the pituitary-restricted POU-homeodomain transcription factor, Pit-1 (POU1-F1) (17,18). HSI actions include the activation of an adjacent domain of noncoding transcription (18,22). HSI enhancer function is directly and quantitatively related to the intensity of this PolII transcription and is independent of the structure of the encoded RNA transcripts (23). In the current report, we demonstrate that HSI can activate PolII-mediated noncoding transcription independent of linked promoter elements (Figure 1) and that the corresponding TTSs can be generated by HSI within multiple DNA sequence contexts (Figures 1 and 3). Importantly, the timing and positioning of this HSI-autonomous activity is tightly linked to activation of the *GH* gene in the embryonic pituitary (Figure 2). These data support a model in which HSI-activation of the adjacent domain of noncoding transcription constitutes an essential and initiating event in the pathway of *hGH-N* gene expression.

How HSI activates its domain of noncoding transcription was addressed by mapping the position of the corresponding TSSs. 5′ RACE studies revealed that the TSSs that define the 5′ terminus of the noncoding transcriptional domain are established at a mean distance of 1.6 ± 0.2 kb from the HSI core elements. This distance was maintained despite the introduction of large deletions in the *hCD79b* gene and by replacing the *hCD79b* gene with a segment of λ-DNA (Figure 3). These data led us to hypothesize that HSI establishes a specific higher-order chromatin configuration in the pituitary that defines a spatial relationship between HSI and its sites of PolII transcriptional initiation.

The 5′ RACE analysis of the intact *hGH BAC* in the pituitary revealed that in addition to the activation of a cluster of TSSs at the conserved spacing of 1.6 ± 0.2 kb, two additional sets of TSSs are generated further 3′ (Figure 3). These additional TSS clusters may reflect enhanced accessibility due to the extensive (32 kb) domain of HSI-generated activating histone modifications encompassing HSV through to the *hGH-N* promoter in the pituitary locus (18). This contrasts with activated domain at the B-cell chromatin locus that is tightly focused over the *hCD79b* promoter (26). While these additional sets of TSSs are not further explored, they serve to highlight the independent pathways and mechanisms of transcriptional initiation within the *hGH* locus in pituitary and B-cell.

Structural parameters that dictate nucleosome positioning and occupancy in the mammalian genome *in vivo* remain incompletely understood. Nucleosomes can be organized in phased arrays or they can lack specific positioning, taking an unstructured or ‘fuzzy’ configuration (33). Determinants of nucleosome phasing include specific DNA motifs that strongly bind histone octamers as well as DNA binding proteins such as certain transcription factors that strongly occupy a specific site (35). Tightly positioned nucleosome can serve as organizing boundaries (‘positional barriers’) against which neighboring nucleosomes are packed (‘statistical positioning’) (36,37). A lack of clearly defined positioning of nucleosomes in other regions may reflect mobile and/or stochastically positioned nucleosomes (33). This unstructured configuration may serve specific functional demands in transcriptional control. Thus, distinct nucleosome patterns and accompanying higher order chromatin configurations that distinguish any two tissues is likely to reflect distinct functional states and cell-type specificities (38). In this respect, the comparison of nucleosome occupancy within the *hGH* locus in pituitary in B cells may be particularly informative.

The comparison of mononucleosome-protected DNA fragments generated from pituitary and B cells of *hGH BAC* transgenic mice reveals strong similarities as well as distinct differences. The chromatin architecture encompassing the *hGH-N* promoter is marked in both tissues by a nucleosome-free regions bracketed by strong peaks of nucleosome occupancy (Figure 5B). A nucleosome-free region was also present in both tissues at a site corresponding to the *hCD79b* promoter (Figure 5C). This type of configuration at gene promoters is well established in genome-wide surveys (39). In contrast, the nucleosome architecture in the region encompassing HSI and its noncoding TSS is markedly divergent between pituitary and B cells (Figure 5C). HSI in the pituitary sample co-maps with a strongly defined nucleosome-free region flanked by positioned nucleosomes. The specificity of a nucleosome-free region at this site in pituitary chromatin is consistent with the corresponding pituitary-specific DNaseI sensitive site (HSI) (26) and its occupancy by the pituitary-specific factor Pit-1 (17). The second singularity at the pituitary chromatin locus is that the region between the HSI and the *hCD79b* promoter comprises a 600-bp region that is occupied by nucleosomes but lacks an organized nucleosomal configuration. While the exact structure of this ‘fuzzy’ domain flanked by a set of strong nucleosome occupancy peaks remains to be further explored (Figure 5C), its lack of structure may allow for a enhanced flexibility and/or folding of the chromatin strand. This localized flexibility, if substantiated, might underlie and define the specified distancing between the HSI and its noncoding TSSs. Subsequent ‘looping’ of the focus of intense PolII activity into close proximity with the *hGH-N* promoter would place the target promoter within a microenvironment that strongly enhances transcriptional activity.

## SUPPLEMENTARY DATA

Supplementary Data are available at NAR Online.

SUPPLEMENTARY DATA

## References

[1] Smallwood A., Ren B. (2013). Genome organization and long-range regulation of gene expression by enhancers. Curr. Opin. Cell Biol..

[2] Dekker J., Rippe K., Dekker M., Kleckner N. (2002). Capturing chromosome conformation. Science.

[3] Sanyal A., Lajoie B.R., Jain G., Dekker J. (2012). The long-range interaction landscape of gene promoters. Nature.

[4] Sexton T., Bantignies T., Cavalli G. (2009). Genomic interactions: chromatin loops and gene meeting points in transcriptional regulation. Semin. Cell Dev. Biol..

[5] Noordermeer D., Branco M.R., Splinter E., Klous P., van Ijcken W., Swagemakers S., Koutsourakis M., van der Spek P., Pombo A., de Laat W. (2008). Transcription and chromatin organization of a housekeeping gene cluster containing an integrated beta-globin locus control region. PLoS Genet..

[6] Bonn S., Zinzen R.P., Girardor C., Gustafson E.H., Perez-Gonzalez A., Delhomme N., Ghavi-Helm Y., Wilczynski B., Riddell A., Furlong E.E.M. (2012). Tissue-specific analysis of chromatin state identifies temporal signatures of enhancer activity during embryonic development. Nat. Genet..

[7] Kireeva M.L., Walter W., Tchernajenko V., Bondarenko V., Kashlev M., Studitsky V.M. (2002). Nucleosome remodeling induced by RNA polymerase II: loss of the H2A/H2B dimer during transcription. Mol. Cell.

[8] Carter D., Chakalova L., Osborne C.S., Dai Y.-F., Fraser P. (2002). Long-range chromatin regulatory interactions *in vivo*. Nat. Genet..

[9] Hatzis P., Talianidis I. (2002). Dynamics of enhancer-promoter communication during differentiation-induced gene activation. Mol. Cell.

[10] Ørom U.A., Derrien T., Beringer M., Gumireddy K., Gardini A., Bussotti G., Lai F., Zytnicki M., Notredame C., Huang Q. (2010). Long noncoding RNAs with enhancer-like function in human cells. Cell.

[11] Kim T.-K., Hemberg M., Gray J.M., Costa A.M., Bear D.M., Wu J., Harmin D.A., Laptewicz M., Barbara-Haley K., Kuersten S. (2010). Widespread transcription at neuronal activity-regulated enhancers. Nature.

[12] Wang K.C., Yang Y.W., Liu B., Sanyal A., Corces-Zimmerman R., Chen Y., Lajoie B.R., Protacio A., Flynn R.A., Gupta R.A. (2011). A long noncoding RNA maintains active chromatin to coordinate homeotic gene expression. Nature.

[13] Jones B.K., Monks B.R., Liebhaber S.A., Cooke N.E. (1995). The human growth hormone gene is regulated by a multicomponent locus control region. Mol. Cell. Biol..

[14] Fleetwood M.R., Ho Y., Cooke N.E., Liebhaber S.A. (2012). DNase I hypersensitive site II of the human growth hormone locus control region mediates an essential and distinct long-range enhancer function. J. Biol. Chem..

[15] Bennani-Baiti I.M., Asa S.L., Song D., Iratni R., Liebhaber S.A., Cooke N.E. (1998a). DNase I-hypersensitive sites I and II of the human growth hormone locus control region are a major developmental activator of somatotrope gene expression. Proc. Natl. Acad. Sci. U.S.A..

[16] Su Y., Liebhaber S.A., Cooke N.E. (2000). Human growth hormone gene cluster locus control region supports position-independent pituitary- and placenta-specific expression in the transgenic mouse. J. Biol. Chem..

[17] Shewchuk B.M., Asa S.L., Cooke N.E., Liebhaber S.A. (1999). Pit-1 binding sites at the somatotrope-specific DNase I hypersensitive sites I, II of the human growth hormone locus control region are essential for *in vivo hGH-N* gene activation. J. Biol. Chem..

[18] Ho Y., Elefant F., Cooke N.E., Liebhaber S.A. (2002). A defined locus control region determinant links chromatin domain acetylation with long-range gene activation. Mol. Cell.

[19] Ho Y., Elefant F., Liebhaber S.A., Cooke N.E. (2006). Locus control region transcription plays an active role in long-range gene activation. Mol. Cell.

[20] Bennani-Baiti I.M., Cooke N.E., Liebhaber S.A. (1998b). Physical linkage of the human growth hormone gene cluster and the *CD79b* (*Igβ*/*B29*) gene. Genomics.

[21] Hermanson G.G., Eisenberg D., Kincade P.W., Wall R. (1988). B29: a member of the immunoglobulin gene superfamily exclusively expressed on beta-lineage cells. Proc. Natl. Acad. Sci. U.S.A..

[22] Cajiao I., Zhang A., Yoo E.J., Cooke N.E., Liebhaber S.A. (2004). Bystander gene activation by a locus control region. EMBO J..

[23] Yoo E.J., Cooke N.E., Liebhaber S.A. (2012). An RNA-independent linkage of noncoding transcription to long-range enhancer function. Mol. Cell. Biol..

[24] Omori S.A., Wall R. (1993). Multiple motifs regulate the B cell-specific promoter of the *B29* gene. Proc. Natl. Acad. Sci. U.S.A..

[25] Yoo E.J., Cooke N.E., Liebhaber S.A. (2013). Identification of a secondary promoter within the human B cell receptor component gene *hCD79b*. J. Biol. Chem..

[26] Yoo E.J., Cajiao I., Kim J.S., Kimura A.P., Zhang A., Cooke N.E., Liebhaber S.A. (2006). Tissue-specific chromatin modifications at a multigene locus generate asymmetric transcriptional interactions. Mol. Cell. Biol..

[27] Nissim S., Allard P., Bandyopadhyay A., Harfe B.D., Tabin C.J. (2007). Characterization of a novel ectodermal signaling center regulating *Tbx2* and *Shh* in the vertebrate limb. Dev. Biol..

[28] Frohman L.A., Downs T.R., Kashio Y., Brinster R.L. (1990). Tissue distribution and molecular heterogeneity of human growth hormone-releasing factor in the transgenic mouse. Endocrinology.

[29] Valouev A., Johnson S.M., Boyd S.D., Smith C.L., Fire A.Z., Sidow A. (2011). Determinants of nucleosome organization in primary human cells. Nature.

[30] Phanstiel D.H., Boyle A.P., Araya C.L., Snyder M.P. (2014). Sushi.R: flexible, quantitative and integrative genomic visualizations for publication-quality multipanel figures. Bioinformatics.

[31] Zhu X., Cleiberman A.S., Rosenfeld M.G. (2007). Molecular physiology of pituitary development: Signaling and transcriptional networks. Physiol. Rev..

[32] Hirzmann J., Luo D., Hahnen J., Hobom G. (1993). Determination of messenger RNA 5′-ends by reverse transcription of the cap structure. Nucleic Acids Res..

[33] Flores O., Deniz O., Soler-Lopez M., Orozco M. (2014). Fuzziness and noise in nucleosomal architecture. Nucleic Acids Res..

[34] Ho Y., Liebhaber S.A., Cooke N.E. (2011). The role of the *hGH* locus control region in somatotrope restriction of *hGH-N* gene expression. Mol. Endocrinol..

[35] Kiyama R., Trifonov E.N. (2002). What positions nucleosomes? A model. FEBS Lett..

[36] Kornberg R.D., Stryer L. (1988). Statistical distributions of nucleosomes: nonrandom locations by a stochastic mechanism. Nucleic Acids Res..

[37] Ioshikhes I.P., Albert E., Zanton S.J., Pugh B.F. (2006). Nucleosome positions predicted through comparative genomics. Nat. Genet..

[38] Jiang C., Pugh B.F. (2009). Nucleosome positioning and gene regulation: advances through genomics. Nat. Rev. Genet..

[39] Schones D.E., Cui K., Cuddapah S., Roh T.Y., Barski A., Wang Z., Wei G., Zhao K. (2008). Dynamic regulation of nucleosome positioning in the human genome. Cell.

