# Supplementary material for: Autonomous actions of the human growth hormone long-range enhancer
Source: Nucleic Acids Res. 2015 Feb 6;43(4):2091–101. doi: 10.1093/nar/gkv093 (PMC4344525; doi:10.1093/nar/gkv093)
Supplement: SUPPLEMENTARY DATA [file supp_43_4_2091__index.html]

Autonomous actions of the human growth hormone long-range enhancer — Autonomous actions of the human growth hormone long-range enhancer — SUPPLEMENTARY DATA 

# Autonomous actions of the human growth hormone long-range enhancer

## SUPPLEMENTARY DATA

**Files in this Data Supplement:**

- SUPPLEMENTARY DATA
- SUPPLEMENTARY DATA
