# Supplementary material for: Autonomous actions of the human growth hormone long-range enhancer
Source: Nucleic Acids Res. 2015 Feb 6;43(4):2091–101. doi: 10.1093/nar/gkv093 (PMC4344525; doi:10.1093/nar/gkv093)
Supplement: SUPPLEMENTARY DATA [file supp_gkv093_nar-03328-v-2014-File003.pdf]

Supplementary Table SI. Oligonucleotides used in this study.

5'-RACE	
hCD79b-1	5'-CCTGCATCTAGATCATGGGGCGACCTGGCT-3'
hCD79b-2	5'-GCTTCTAGAGCCAGCTCACATTGCCGGAGG-3'
lambda-1	5'-GCAAACGCAAGGATTGCCCCGATGCC-3'
lambda-2	5'-GTTCCTTTGCCGCGAGAATGGC-3'
Adapter-polyC$_{17}$,	5'-CATCGGACTCGAGTCGAACCCCCCCCCCCCCCCCC-3'
Adapter	5'-CATCGGACTCGAGTCGAA-3'
in situ Hybridization	
mPit-1 5'	5'-GTCATTATGGAAACCAGCCATCC-3'
mPit-1 3'	5'-AGCTACACTGATGGTTGTCC-3'
mGH1 5'	5'-CCTACAAAGAGTTCGAGCGTGCCTAC-3'
mGH1 3'	5'-CACTTCATGACCCGCAGGTAGGTCTC-3'
hCD79b 5'	5'-CAGCGCCTCCGGCAATGTGAGCTGGC-3'
hCD79b 3'	5'-TCATGGGGCGACCTGGCTCTCACTCC-3'
Library Preparation	
MP Adaptor 1	5'-[Phos]GATCGGAAGAGCACACGTC*T-3'
MP Adaptor 2	5'-ACACTCTTTCCCTACACGACGCTCTTCCGATC*T-3'
MP PCR 1	5'-AATGATACGGCGACCACCGAGATCTACACTCTTTCCCTACACGACGCTCTT CCGATC*T-3'
MP PCR 2 index 2	5'-CAAGCAGAAGACGGCATACGAGATACATCGGTGACTGGAGTTCAGACGTG TGCTCTTCCGATC*T-3'
MP PCR 2 index 4	5'-CAAGCAGAAGACGGCATACGAGATTGGTCAGTGACTGGAGTTCAGACGTG TGCTCTTCCGATC*T-3'
MP PCR 2 index 5	5'-CAAGCAGAAGACGGCATACGAGATCACTGTGTGACTGGAGTTCAGACGTG TGCTCTTCCGATC*T-3'
MP PCR 2 index 6	5'-CAAGCAGAAGACGGCATACGAGATATTGGCGTGACTGGAGTTCAGACGTG TGCTCTTCCGATC*T-3'
MP PCR 2 index 7	5'-CAAGCAGAAGACGGCATACGAGATGATCTGGTGACTGGAGTTCAGACGTG TGCTCTTCCGATC*T-3'
MP PCR 2 index 12	5'-CAAGCAGAAGACGGCATACGAGATTACAAGGTGACTGGAGTTCAGACGTG TGCTCTTCCGATC*T-3'

* Phosphorothioate bond
