# Supplementary material for: Autonomous actions of the human growth hormone long-range enhancer
Source: Nucleic Acids Res. 2015 Feb 6;43(4):2091–101. doi: 10.1093/nar/gkv093 (PMC4344525; doi:10.1093/nar/gkv093)
Supplement: SUPPLEMENTARY DATA [file supp_gkv093_nar-03328-v-2014-File004.pdf]

```
Lambda       1 GTTTCGGGATTTTTTA----TATCTGCACAACAGGTAAGAGCA----TTG  42
CD-1.6       1 CAGCCCAGTGAAAGGAGG--GGCCAGGCCTG-TGG-GCCTCCAGGTCCTG  46
CD-0.7       1 CCTCCTGTCTCATTCAGA--TGCCTGCTCAA-AGCCACCATGAGGGTCAA  47
CD           1 CAGACGGCAGAGGGGAGGCTGGCTGGCCCAG-GGG-ATGACCA----CCG  44
                   *          *         *  *    *        *

Lambda      43 AGTCGATAATCGTGAAGAG-TCGGCGAGCCTGGTTAGCCAGTGCTCTTTC  91
CD-1.6      47 GGGGTGTGGCCTGGGTCAGGCTGTTGTAATGGG--CATAGGAAGGAATGG  94
CD-0.7      48 AGGCAGAAAGGGGGCTGGGGGCAGGGCTTCCGGTACCCCCACCCCA-TAT  96
CD          45 GTGGGGTAAGCACAGACAGAGGGGAGCACAGGCTTCCCCCAGAAGACTGA  94
                                 *      *     *               *

Lambda      92 CGTTGTGCTGAATTAAGCGAATACCGGAAGCAGAACCGGATCACCAAATG 141
CD-1.6      95 CAAGGTCCCAGGGCTCTTCCAAAGGAAAATCGAAGGGGAGGTGGGGCAGT 144
CD-0.7      97 CTGTCTCCCAAGTACTGGGGGCAGGGAGGACCGAGGGGTCTTGAAGCTGC 146
CD          95 GAGGCCCCCCAGAGGCATCCACAG-AGGACCCCAGCTGTGCTGCCCAAGC 143
                      *              *       *  *   *

Lambda     142 CG----TACAGGCGTCATCGCCGCCCAGCAACAGCACAACCC--AAACTG 185
CD-1.6     145 CG----GGAAGAGGTGG--AGT-CTGAG-AGCTTGGCCGT----GGCTTC 182
CD-0.7     147 TGTCATGGAAGGAGGAAGTAGC-TCCGGGAACATAGAGGGCATGCAGGGG 195
CD         144 TG----GGCGACCGCCA--AAC-CTTAGCGGCCCAGCTGACA--AAAGCC 184
                *           *             *   *

Lambda     186 AGCCGTAGCCACTGTCTGTCCTGAATTCATTAGTAATAGTTACG-CTGCG 234
CD-1.6     183 TCATGTGGCT-CAGCCTCTCACGTGGCTCTGCTTCCCAACTCCAACCTCA 231
CD-0.7     196 TGGGGCAGCC-CAGGC-CAGAGGAGGCCCTGA---CTGGCCCCGCCCACT 240
CD         185 TGCCCTCCCC-CAGGGTCCC-CGGAGAGCTGGTGCCTCCCCTGGGTCCCA 232
                       *  * *        *      *                  *

Lambda     235 GCCTTTTACACATGACCTTCGTGAAAGCGGGTGGCAGGAGGTCGCGCTAA 284
CD-1.6     232 GCCTCC-TGCCTGTCCCACACCCTGAGAAGCAAGGACTAAGCCCAGGGAG 280
CD-0.7     241 TCCTCCCACCCAGCAGCTGAGCCAGT--ACCAGCAGCCAGATC-GGAGGA 287
CD         233 ATTTGCATGGCAGGAAGGGGCCTGGTGAGGAAGAGGCGGGG---AGGGGA 279
                  *      *                                  *

Lambda     285 CAACCTCCTGCCGTTT----- 300
CD-1.6     281 TCTGTGCCAGCCAGGGCAGG- 300
CD-0.7     288 CCGGTACCGG--AAT------ 300
CD         280 CAGGCTGCAGCCGGTGCAGTT 300
                      * *
```

Supplementary Figure S1. The four sets of sequences correspond to the TSS clusters mapped 3' to HSI in the pituitaries of mice carrying each of the indicated transgenes. The aligned sequences lack TATA boxes, CpG rich domains, or conserved primary sequence motifs that might account for Pol II TSS positioning. Each 300 bp sequence in the alignment was positioned at the 50 bp window with the highest frequent TSS (**Figure 3**) and its 250 bp 5' flanking region. The asterix indicates sites of full sequence identity. The multiple alignment was performed using ClustalW (v1.83). Lambda indicates the sequence from λ gene of *λΔCD/hGH BAC*; CD-1.6, sequence from the major TSS of *CDΔ1.6/hGH BAC*; CD-0.7, sequence from the first major TSS of *CDΔ0.7/hGH BAC*; CD, sequence from the first major TSS in promoter of *hCD79b* of *hGH BAC*. The promoter of *hCD79b* has been previously reported as a TATA-less promoter (Thompson et al., 1996, Blood, 87:666-673).
